# Effectiveness of the Functional and Cognitive Occupational Therapy (FaC_o_T) Intervention for Improving Daily Functioning and Participation of Individuals with Mild Stroke: A Randomized Controlled Trial

**DOI:** 10.3390/ijerph18157988

**Published:** 2021-07-28

**Authors:** Tal Adamit, Jeffrey Shames, Debbie Rand

**Affiliations:** 1 Department of Occupational Therapy, Sackler Faculty of Medicine, Tel Aviv University, Tel-Aviv 6997801, Israel; 2 Maccabi Health-Care Services, Tel-Aviv 6812509, Israel; Sheimes_j@mac.org.il

**Keywords:** stroke rehabilitation, cognitive–functional interventions, PROMs, participation

## Abstract

Background: Mild stroke can cause subtle cognitive–behavioral symptoms, which although might be hidden, can restrict community reintegration and participation. Cognitive rehabilitation programs exist for stroke but not specifically for mild stroke and the research evidence varies. The Functional and Cognitive Occupational Therapy (FaC_o_T) intervention was developed specifically for this population. Objective: To examine the effectiveness of FaC_o_T intervention for improving daily functioning and participation compared with standard care. Method: A single blind randomized controlled trial with assessments pre (T1), post (T2) and 3-month follow-up (T3). Individuals in the FaC_o_T group received 10 weekly sessions practicing cognitive and behavioral strategies. The Canadian Occupational Performance Measure (COPM) was the primary outcome measure, IADL-questionnaire, Reintegration to Normal Living questionnaire (RNL) were secondary measures. Results: In total, 66 community-dwelling individuals with mild stroke were randomly allocated to FaC_o_T (*n* = 33, mean (SD) age 64.6 (8.2), 33% women), or control group (*n* = 33, mean (SD) age 64.4 (10.8), 45% women). Time X Group interaction effects were found for the COPM performance (*F*(1.4,90.3) = 11.75, *p* < 0.000) and satisfaction (*F*(1.5,96.8) = 15.70, *p* < 0.000), with large effect size values. Significant between-group effects were found for RNL (*F* = 10.02, *p* < 0.002, *ɳ_P_^2^* = 0.13). Most participants in FaC_o_T achieved a clinically important difference in COPM between T1–T2, T1–T3, and in RNL between T1 to T3 compared with the control group. Conclusions: FaC_o_T intervention is effective to improve daily functioning, participation and satisfaction of individuals with mild stroke compared with standard care, therefore FaC_o_T should be implemented in community rehabilitation settings.

## 1. Introduction

Despite the high prevalence of mild stroke, very few studies have focused on this subgroup. A stroke is considered “mild” when neurological deficits (such as muscle weakness or dysarthria) are minimal, or Basic Activities of Daily Living (BADL) (dressing, bathing) is only minimally affected [1,2,3]. Therefore, mild strokes can be easily overlooked and many individuals do not benefit from rehabilitation services after acute care hospitalization [4,5]. These individuals experience difficulties in Instrumental Activities of Daily Living (IADL) (such as shopping or driving), productivity and leisure [6]. Participation, is considered involvement in life situations [7], is an important indicator of human health and well-being [8]. Low levels of participation, restricted family and social involvement, and difficulties re-integrating into meaningful occupations [9,10] have been reported, leading to decreased satisfaction and low quality of life detected even up to 18-months post mild stroke [9,11,12]. 

These difficulties are presumably due to the subtle, and often hidden, cognitive and emotional–behavioral symptoms [5,13]. Cognitive deficits include mainly deficits in higher cognitive abilities [14,15], termed executive functions, such as planning, decision making and self-control. Executive functions are crucial for performing complex daily functions, especially in novel situations such as shopping, driving and working [16]. The behavioral–emotional symptoms can include irritability, apathy, difficulty expressing emotions, response inhibition and restlessness [2,13]. Individuals with mild stroke may also experience low self-efficacy [17], which is a decreased belief in their own ability to perform premorbid significant occupations [18]. Low self-efficacy has been associated with depression, disability and decreased quality of life, assessed 6-months post mild stroke [19,20].

Cognitive–functional occupational therapy interventions, which do not focus on improving specific cognitive deficits but rather on improving daily activities, have been developed for individuals with traumatic brain injury and stroke [21,22,23]. The Multicontext Approach [24] and The Cognitive Orientation to Daily Occupational Performance (CO-OP) [25] for example, include teaching individuals to use cognitive strategies to improve daily activities (termed occupational performance). Cognitive strategies are tools, procedures or methods used to accomplish goals [26]. Clinical studies, which tested the effectiveness of these interventions, have demonstrated improvement in occupational performance of individuals with stroke [25] (not mild stroke) and traumatic brain injury [27] but samples are very small, follow-up periods are short and research evidence is limited. In addition, these interventions do not address the unique behavioral-emotional changes, which are apparent in addition to the executive function deficits after mild stroke. 

The Functional and Cognitive Occupational Therapy (FaC_o_T) intervention (pronounced—FACT), theoretically anchored [28,29,30,31,32] was developed to focus on the unique characteristics of individuals with mild stroke, specifically experiencing executive function deficits and emotional-behavioral symptoms [33,34]. FaC_o_T includes practicing the use of cognitive and behavioral strategies to overcome these deficits/symptoms. This study aimed to examine the effectiveness of FaC_o_T to enhance occupational performance and participation of individuals with mild stroke compared with standard care. 

## 2. Materials and Methods 

### 2.1. Participants

A single-blind-randomized controlled trial (RCT) with assessments pre (T1) and post (T2) intervention and at 3-month follow-up (T3) were conducted by assessors (experienced occupational therapists) who were blind to group allocation. This trial followed the Consolidated Standards for Reporting Trials (CONSORT) statement for Randomized Trials [35,36] and was registered in ClinicalTrials.gov (accessed on 20 July 2021) registration (NCT02925637). The study was approved by the Helsinki committee and University Ethics committee, and all participants provided written informed consent before their participation.

Community-dwelling individuals with mild stroke were recruited from a community-based Health Care Service between March 2017 and February 2020. Inclusion criteria were: (1) age >18 years, (2) sustained a stroke in the last 36 months, (3) mild stroke severity [National Institutes of Health Stroke Scale (NIHSS) ≤ 5 points and/or independence in BADL], (4) independence in daily living prior to the stroke, (5) ability to understand and speak the language (Hebrew), (6) without other neurological or psychiatric conditions. Sample size was calculated based on the primary outcome measure in G-Power analyses for *F*-test ANOVA repeated measures with 80% power and significance level of 0.05. To account for 15% dropout, at least 30 participants per group had to be recruited.

### 2.2. Randomization

Participants were approached by phone and eligible participants were invited for the assessment session (T1). Following T1, stratified random sampling was performed using a block (6) randomization procedure (ratio 1:1). Participants were stratified into either higher (≥23 points) or lower (≤22 points) cognitive status using the Montreal Cognitive Assessment (MoCA) [37], a valid and reliable [38] cognitive screening tool. Participants were informed their allocation by phone.

### 2.3. Outcome Measures

Patient-Reported Outcome Measures (PROMs) [39] were used to quantify participant’s perception of their functional status. The primary outcome measure aimed to assess occupational performance (daily functioning). We used the Canadian Occupational Performance Measure (COPM) [40], which is a well-known occupational therapy tool, commonly used for interventional studies [25,41], valid and reliable for people with stroke [42]. This semi-structured interview is used to identify problems and detect change over time in performance and satisfaction of daily occupations. Participants defined four goals (such as performing regular physical activity, meeting with friends) and for each goal, using a 10-point visual analogue scale [from 1 (low) to 10 (excellent)] rated their performance and satisfaction from performance. Two of the four goals were chosen to be the focus of the intervention. At T2 and T3 the same four occupational goals defined at T1 were re-rated. A change of 2 points or more is considered a minimal clinically important difference (MCID) [43].

Participation and independence in instrumental activities of daily living were considered secondary outcome measures. The Reintegration to Normal Living index (RNL) [44], is a self-report questionnaire to evaluate reintegration to productive, social and leisure activities. The total score ranges from 11 to 110, and then proportionately transformed to 100 points, a higher score indicates more reintegration into the community. Normative data and data of stroke patients have been published [45]. A change in the total score of at least 14.8 points is considered a minimal detectable change (MDC) [46]. Independence in IADL was assessed by the IADL questionnaire (IADLq) [47], which assesses eight domains of IADL such as housekeeping, meal preparation and shopping. The total score is the sum of all the items from 0 (dependent) to 23 (independent). A change of 3 points or more is considered a minimal detectable change (MDC) [48] for the IADLq. 

Demographic (age, sex, education, pre-morbid function) and stroke information (side, type of lesion, referral to rehabilitation, subacute or chronic stage since stroke onset) was collected.

### 2.4. Intervention

The experimental group received FaC_o_T; 10 1-h weekly individualized sessions with an experienced OT. Sessions included task analysis of two of the participant’s occupational performance goals (as defined by the COPM, such as meeting a friend for coffee) to identify the specific difficulty within the goal (i.e., initiating and making the arrangement, planning the use of transportation to arrive on time). Then the OT taught and practiced the use of cognitive and behavioral strategies via case studies tailored to the participant’s goals. Cognitive strategies focused on different components of executive functioning: Initiation, Inhibition, Planning and Decision-Making strategies. The behavioral strategies included practicing the following strategies: Self-perception (in terms of ability, competency and efficacy), Situation interpretation and Future prediction. Practicing of strategies was done by analyzing case studies using the point-of-view of two personas (one positive and the other negative). FaC_o_T also provided knowledge to increase awareness regarding symptoms of mild stroke such as fatigue and hidden symptoms (not visible to others and not identified by the participants themselves). The use of positive therapeutic language and positive feedback to raise awareness, improve self-efficacy and promote the transfer of the strategies to different daily activities was integrated in the FaC_o_T sessions. The OTs who carried out the intervention filled in a fidelity checklist following each session and kept a log of the participant’s comments and reactions.

The control group, who underwent the same cognitive–functional assessments, including identification of four meaningful occupational performance goals by COPM, received standard care. 

### 2.5. Statistical Analysis

Descriptive statistics were used to describe the groups and the dependent variables at T1, T2 and T3 and compare between groups pre-intervention using *t*-test for independent samples or chi-square test for dichotomous measures. To test the effectiveness of FaC_o_T compared to control groups at T1, T2 and T3, a repeated measures 2(groups) X 3(time) analysis of variance ANOVA was used to compare within and between group scores, and for Time X Group interaction effect. Mauchly’s Test of Sphericity was used, and the Greenhouse–Geisser procedure was conducted to correct the degrees of freedom. The magnitude of the difference was calculated by Partial Eta squared; 0.01, 0.06 and 0.14 values were considered small, medium and large effect size, respectively [49]. Main effects of time were interpreted by a post-hoc pairwise comparison with the Bonferroni correction. To understand the group effects, *t*-test for independent samples with Cohen’s *d* were performed for the RNL scores at T2 and T3. Percent change of the outcome measures in each group between T1–T2 and between T1–T3 were calculated using this formula $\left[\frac{T2\;\mathrm{or}\;T3-T1}{T1}\times 100\right]$. Chi-square test was used to assess between-group differences regarding the percentage of participants who achieved the MCID for the COPM performance and satisfaction and the MDC for the IADLq and RNL at T2 and T3. Data was analyzed with SPSS statistical software version 26 using an intention-to-treat analysis [50] including all randomized data, with the last point carried forward.

## 3. Results

**Figure 1:**
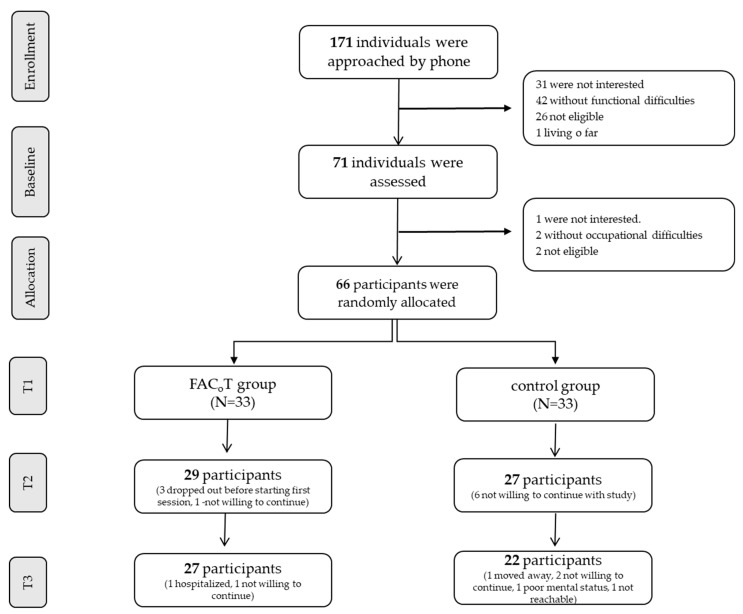
CONSORT flow diagram (Note: at T2 and T3, all 33 cases in each group were analyzed using intention-to-treat analysis).

In total, 171 individuals with mild stroke were approached and screened by phone. Of them, 71 individuals were invited to T1; 66 individuals were eligible and were enrolled into the study (See Figure 1 for the CONSORT flow diagram). A total of 33 individuals were allocated into FaC_o_T group (11 women; mean (SD) age 64.6 (8.2); 85% of them were in the chronic stage, MoCA 21.5 (3.9) points) and 33 individuals into the control group (15 women; mean (SD) age 64.4 (10.8); 79% of them in chronic stage, MoCA 21.8 (4.1) points) (see Table 1). As can be seen in Table 1, groups were similar at baseline.

Most participants from both groups had sustained a first stroke and demonstrated cognitive deficits. Participants were independent in BADL, but experienced difficulties in IADL, return to work and participation restriction. At T1, the primary [COPM (performance (t = −1.7, *p* = 0.1), satisfaction (t= −1.7, *p* = 0.1)] or secondary outcome measures were not significantly different between groups (*p* > 0.05).

In total, 29 participants from the FaC_o_T group completed the intervention and were assessed at T2, and 27 of them completed T3. The low dropout rate was seen in the control group as well (Figure 1).

Mauchly’s Test of Sphericity indicated that the assumption of sphericity had been violated for COPM performance (χ^2^ (2) = 42.63, *p* = 0.000) and satisfaction (χ^2^ (2) = 33.99, *p* = 0.000) and the Greenhouse–Geisser correction to the degrees of freedom was completed. Significant within-subject effects were found for COPM performance (*F*(1.4,90.33) = 48.44, *p* = 0.000, *ɳ_P_^2^* = 0.43) and satisfaction (*F*(1.5,96.8) = 53.53, *p* < 0.000, *ɳ_P_^2^* = 0.46) with large effect sizes values. Post hoc analysis with a Bonferroni adjustment revealed that COPM performance and satisfaction were statistically significantly increased from T1 to T2 (−1.94 (95% CI, −2.6 to −1.3), *p* = 0.000; −2.22 (95% CI, −2.9 to −1.5), *p* = 0.000, respectively) and T1 to T3 (−2.16 (95% CI, −2.9 to −1.4), *p* = 0.000; −2.6 (95% CI, −3.4 to −1.8), *p* = 0.000, respectively). 

The Time X Group effect was significant for performance (*F*(1.4,90.3) = 11.75, *p* < 0.000) and satisfaction (*F*(1.5,96.8) = 15.70, *p* < 0.000) with large effect size values from T1 to T2 (performance *ɳ_P_^2^* = 0.19; satisfaction *ɳ_P_^2^* = 0.23) and T1 to T3 (performance *ɳ_P_^2^* = 0.16; satisfaction *ɳ_P_^2^* = 0.23) (See Table 2). In addition, the percent improvement of the COPM was very large for the FaC_o_T compared to the control group, especially for the COPM satisfaction. Between T1 and T2, the percent improvement for FaC_o_T was 207.4% and 241% compared to 39.7% and 55.6% for the control for COPM performance and satisfaction (respectively) (See Table 3). Between T1 and T2, 61% of the participants in FaC_o_T and 15% of the participants in the control group achieved the MCID for the COPM performance, which was significant (*χ*^2^ = 14.50, *p* < 0.0001), and 70% compared with 24% of the participants achieved the MCID for COPM satisfaction (*χ*^2^ = 13.69, *p* < 0.000). Between T1 to T3, 61% and 63% of the participants in FaC_o_T (versus 18% and 31%) achieved a MCID for COPM performance and satisfaction (*χ*^2^ = 12.44, *p* < 0.0001, *χ^2^* = 8.15, *p* < 0.004, respectively). 

Smaller percent improvements were seen for the secondary outcome measures (See Table 3). Significant within-subject effects with Greenhouse-Geisser correction were found for IADLq (*F*(1.6,103.6) = 6.42, *p* < 0.002, *ɳ_P_^2^* = 0.09), and RNL (*F*(1.8,114.3 = 5.44, *p* < 0.005, *ɳ_P_^2^* = 0.08), with medium effect sizes. IADLq and RNL were statistically significantly increased from T1 to T2 (−1.38 (95% CI, −2.6 to −0.2), *p* = 0.022; −5.33 (95% CI, −9.2 to −1.4), *p* = 0.004, respectively) and also IADLq was statistically significantly increased from T1 to T3 (−1.33 (95% CI, −2.5 to −0.2), *p* = 0.021). Significant between-group effects were found for RNL (*F* = 10.02, *p* < 0.002, *ɳ_P_^2^* = 0.13). Independent *t*-test showed differences with large effect sizes between groups at T2 (t(64) = 3.2, *p* = 0.002, Cohen’s *d* = 0.78) and T3 (t(64) = 3.6, *p* = 0.001, Cohen’s *d* = 0.88). FaC_o_T group had greater improvement at T2 and T3 (mean (SD)- 80.77 (18.22), 79.86 (13.97), respectively) compared to control group (mean (SD)- 66.23 (18.85), 64.98 (19.46), respectively). The Time X Group effects were not significant for the secondary measures. Between T1 to T3, 36.4% of the participants in FaC_o_T achieved MDC for RNL compared to 15.2% participants in the control group, which was significant (χ^2^ = 3.88, *p* < 0.049). No differences were found between groups in the percent of participants who achieved MDC for IADLq.

## 4. Discussion

Individuals with mild stroke are often overlooked and do not receive rehabilitation services despite their deficits [51]. This single-blind randomized controlled trial, aimed to test the effectiveness of FaC_o_T, a novel occupational therapy intervention developed for this population with unique characteristics, for improving occupational performance and participation. Significant findings with a large effect size and a high percentage of participants achieving meaningful clinical changes were found for the FaC_o_T compared with the control group.

The characteristics of the 66 participants with mild stroke who participated in this RCT are in accordance with previous reports of this population [9,33], which supports the external validity of this study. In total, 88% of our participants completed FaC_o_T intervention and 81% were assessed on follow-up, indicating that participants perceived the positive effects of FaC_o_T. Interesting, 82% of the control group returned for assessments at T2 and 66% at T3. Possibly, since participation in the study provided them with a comprehensive cognitive and functional evaluation at each time-point, they were motivated to return. The low dropout from our study groups (FaC_o_T (12%) and control (18%)) is considerably lower than reported for similar interventions including individuals with stroke (CO-OP (63%) and standard occupational therapy (55%)) [25]; Virtual reality training (23%) and control (16%) [52]; CO-OP (17%) and Computer training (20%) [53]. This too might indicate that individuals with mild stroke (as opposed to stroke) are more motivated since they are longing for community rehabilitation services to help them return to their premorbid activities.

Post intervention, the improvement seen in the FaC_o_T group compared with the control group was measured by the COPM, which is a PROM [40], and commonly used in studies post stroke [25,54,55]. PROMs are considered to objectively measure the person’s own management progress and therefore have become very important when assessing the effectiveness of interventions. In addition, the clinical significance analysis of FaC_o_T was done by calculating the effect size and minimal clinical differences, rather than only using statistical significance [56]. Findings regarding MCID allow clinicians to select the most appropriate intervention resulting in clinically significant change [57,58]. The extremely high percent improvement in COPM satisfaction in the FaC_o_T group is possibly due to practicing behavioral strategies, which might have led to improvement in self-efficacy. At T2, 61% of the participants in FaC_o_T (as opposed to 15% in the control) achieved at least a 2-point increase, which is the COPM’s MCID [57], and this trend was maintained at T3. Additionally, the improvement in performance and satisfaction at 3-month follow-up, emphasizes that these participants continued to implement the cognitive and behavioral strategies in their daily living, resulting not only in sustained status but also continued improving.

Significant improvements in participation in the community were found for FaC_o_T compared with control group at T2 and T3, with large effect size values. In the FaC_o_T group 48.5% and 42.4% at T2 and T3 achieved the normal range of participation, respectively [46] compared to only 21.2% at T1. In the control group a reverse trend was seen; a decrease in the percentage of participants who re-integrated to community within the normal range (15.2% at T1, 12.1% at T2, 12.1% at T3) [46]. Therefore, FaC_o_T was effective in promoting participation in individuals with mild stroke indicating that successful rehabilitation has occurred [59]. Participation is not usually assessed as a research outcome measure [59,60] and indeed there is limited research which focuses on improving participation and community reintegration (with no studies found for mild stroke).

Our main limitation is that FaC_o_T was compared to a control group, who received standard care. However, this population of mild stroke receives very little rehabilitation, and this study emphasizes the need to provide this population with some type of intervention. Some improvement was seen in the control group, which might have occurred due to the fact that participants (as part of the three-point evaluation), raised occupational goals, which might have directed attention, awareness and increased their motivation for change. However, the improvement in the control group was not statistically or clinically meaningful.

Our sample was heterogeneous in terms of time since mild stroke because it was difficult to find and recruit individuals who were not receiving rehabilitation. The fact that their cognitive-executive functioning deficits were still apparent at the chronic stage emphasizes the need to provide intervention. The follow-up period of 3-months is relatively short. Future studies should assess if the positive findings remain for longer periods.

## 5. Conclusions

FaC_o_T is effective to improve and promote further improvement of occupational performance, participation and involvement in the community of individuals with mild stroke compared with standard care. FaC_o_T should be implemented in community rehabilitation settings and offered to individuals with mild stroke who have difficulties returning to their premorbid status and are longing for intervention.

## Figures and Tables

**Table 1:** Demographic, functional and stroke-related characteristics of participants in both groups at T1.

	FaC_o_T Group (*n* = 33)	Control Group (*n* = 33)	Differences between Groups
	Mean (SD) Min–Max	Mean (SD) Min–Max	*t*-Test t(*p*) *
Age (years)	64.6 (8.2) 49–77	64.4 (10.8) 48–84	0.1 (0.9)
Education (years)	12.1 (1.9) 8–16	12.9 (2.8) 6–20	−1.2 (0.2)
NIHSS (0–46)	1.2 (1.2) 0–4	1.7 (1.6) 0–6	−1.4 (0.2)
FIM (18–126)	118.8 (7.2) 98–126	117.2 (7.1) 96–126	0.9 (0.4)
MoCA (0–30)	21.5 (3.9) 11–29	21.8 (4.1) 14–28	0.2 (0.8)
	***n* (%)**	***n* (%)**	**Chi-square χ^2^ (*p*)**
Sex F	11 (33.3)	15 (45.5)	1.0 (0.3)
First stroke	20 (60.6)	19 (57.6)	0.1 (0.8)
Stroke side R/L	13/18 (39.4/54.5)	14/16 (42.4/48.5)	0.14 (0.7)
Type of stroke—ischemic/hemorrhage	32/0 (100/0)	31/2 (93.9/6.1)	2.0 (0.2)
Lesion—cortical/subcortical	9/17 (27.3/51.5)	8/19 (24.2/57.6)	0.09 (0.8)
Chronic stage	28 (84.8)	26 (78.8)	0.4 (0.5)
Received Inpatient rehabilitation	5 (15.2)	8 (24.2)	0.87 (0.3)
Received Outpatient rehabilitation	13 (39.4)	9 (27.3)	0.32 (0.6)
Returned to work since stroke (Participants who worked prior to the stroke)	55% (*n* = 11/20)	31% (*n* = 4/13)	1.87 (0.2)

NIHSS—National Institutes of Health Stroke Scale; FIM—Functional Independence Measure; MoCA—Montreal Cognitive Assessment; * degrees of freedom for all *t*-test comparisons (*df* = 64).

**Table 2:** The mean (SD) scores of the PROMs, and Time X Group interaction effect.

	FaC_o_T Group (*n* = 33) Mean (SD) Min–Max			Control Group (*n* = 33) Mean (SD) Min–Max			Time X Group Effect		
	T1	T2	T3	T1	T2	T3	F (df)	*p*	Partial Eta²
COPM performance (0–10)	3.1 (1.3) 1–5	6.0 (2.2) 2–10	6.2 (2.4) 1–10	3.7 (1.3) 0–8	4.7 (2.3) 1–10	4.7 (2.3) 0–10	11.75 (1.4, 90.3)	0.000	0.15
COPM satisfaction (0–10)	2.4 (1.3) 1–6	5.9 (2.3) 1–10	6.4 (2.6) 1–10	3.1 (2.1) 0–9	4.1 (2.5) 1–10	4.4 (2.5) 0–10	15.70 (1.5, 96.8)	0.000	0.20
IADLq (0–23)	17.7 (4.7) 8–23	19.6 (4.2) 7–23	19.6 (4.8) 7–23	17.6 (18.4) 6–23	18.4 (4.4) 7–23	18.3 (4.0) 7–23	1.21 (1.6, 103.6)	0.3000	0.02
RNL (0–100)	72.1 (14.5) 43–100	80.8 (18.2) 20–100	79.9 (13.9) 54–100	64.2 (21.1) 20–100	66.2 (18.8) 25–95	64.9 (19.5) 27–100	2.60 (1.8, 114.3)	0.078	0.04

COPM—Canadian Occupational Performance Measure; IADLq—Instrumental Activities of Daily Living; RNL—Reintegration to Normal Living index.

**Table 3:** The mean (SD) percent change of the measures of both groups between T1 to T2 and T1 to T3.

	FaC_o_T Group (*n* = 33) Mean (SD)		Control Group (*n* = 33) Mean (SD)	
	% Change T1–T2	% Change T1–T3	% Change T1–T2	% Change T1–T3
COPM performance	138.6 (144.9)	153.2 170.9)	39.7 (124.6)	53.0 (133.5)
COPM satisfaction	207.4 (205.8)	241.0 (248.4)	55.6 (127.2)	88.4 (161.9)
IADLq	15.3 (29.8)	15.0 (32.7)	13.5 (51.4)	11.2 (940.3)
RNL	12.6 (22.2)	13.7 (24.1)	7.3 (25.6)	6.9 (32.9)

COPM—Canadian Occupational Performance Measure; IADLq—Instrumental Activities of Daily Living; RNL—Reintegration to Normal Living index.

## Data Availability

The data presented in this study are available on request from the corresponding author. The data are not publicly available due to the immunity of patients’ medical information.
